# Randomised study of the effects of sense of entitlement and conflict of interest contrarianism on researcher decision-making to work with the alcohol industry

**DOI:** 10.1186/s12889-024-18961-5

**Published:** 2024-06-24

**Authors:** Jim McCambridge, Kypros Kypri, Jan R. Boehnke, Lisa Bero, Marcus Bendtsen

**Affiliations:** 1 University of York, Seebohm Rowntree Building, York, YO10 5DD UK; 2 University of Newcastle, Newcastle, Australia; 3 School of Health Sciences, University of Dundee, Dundee, UK; 4 University of Colorado, Aurora, CO US; 5 Department of Health, Medicine and Caring Sciences, Linköping University, Linköping, Sweden

**Keywords:** Conflict of interest, Contrarianism, Entitlement, Industry funding, Alcohol industry

## Abstract

**Background:**

It is well established that the tobacco industry used research funding as a deliberate tactic to subvert science. There has been little wider attention to how researchers think about accepting industry funding. We developed, then tested, hypotheses about two psychological constructs, namely, *entitlement* and *conflict of interest contrarianism (CoI-C)* among alcohol researchers who had previously received industry funding.

**Methods:**

A mixed-methods pilot study involved construct and instrument development, followed by an online survey and nested 3-arm randomised trial. We randomly allocated alcohol industry funding recipients to one of three conditions. In two experimental conditions we asked participants questions to remind them (and thus increase the salience) of their sense of *entitlement* or *CoI-C*. We compared these groups with a control group who did not receive any reminder. The outcome was a composite measure of openness to working with the alcohol industry.

**Results:**

133 researchers were randomised of whom 79 completed the experiment. The posterior distribution over effect estimates revealed that there was a 94.8% probability that reminding researchers of their CoI-C led them to self-report being more receptive to industry funding, whereas the probability was 68.1% that reminding them of their sense of entitlement did so. Biomedical researchers reported being more open to working with industry than did psychosocial researchers.

**Conclusion:**

Holding contrarian views on conflict of interest could make researchers more open to working with industry. This study shows how it is possible to study researcher decision-making using quantitative experimental methods.

**Supplementary Information:**

The online version contains supplementary material available at 10.1186/s12889-024-18961-5.

## Background

Tobacco industry funded research organisations, such as the Council for Tobacco Research, were particularly important in efforts to subvert science, as shown in analyses of tobacco company internal documents [1–3]. The 1998 Tobacco Master Settlement Agreement reached with the tobacco companies required these organisations to be disbanded [4]. Evidence from the tobacco industry documents archive shows that one part of the alcohol industry, representing distilled spirits in the USA, developed similar strategies to subvert science [5]. In fact, distilled spirits companies engaged the services of public relations company Hill and Knowlton before the tobacco companies did [5]. Accepting research funding from alcohol industry actors was commonplace for decades [6–9].

This began to change in the early 1990s as health researchers expressed their concerns about alcohol industry influence on research and policy [10, 11]. As awareness of the tobacco conspiracy grew [12], alcohol researchers drew parallels with the risks industry involvement posed to science and public policy relating to alcohol [13–15]. Scientists strongly articulated their concerns in peer-reviewed journals, particularly as the increasingly globalized alcohol industry came to regard public health science as a threat to its profits [16–19]. By establishing the International Center for Alcohol Policies in the mid-1990s, the alcohol industry increased the scale, reach and sophistication of its efforts to influence science to shape public policy [16, 20, 21].

The alcohol research community was, however, slow to turn its attention to industry efforts to subvert science [22–24]. The subject has engendered controversies periodically within the research community (see for example [25–30]), without resolving them in ways informed by scientific evidence. Polarised views were articulated ‘for’ or ‘against’ alcohol industry research funding [7].

We are aware of no quantitative research concerning how alcohol researchers make decisions about whether to accept funding from industry sources, and there is modest evidence only from studies of the pharmaceutical industry. In an experiment involving medical doctors, Sah and Loewenstein found that reminding doctors of the sacrifices they made in undertaking their training increased their willingness to accept gifts from pharmaceutical companies [31]. They suggested doctors developed a sense of entitlement, the consequence of a self-serving bias which offset reservations they might have about conflict of interest (CoI).

We followed their model for study, and posited that sense of entitlement experimentally manipulated by Sah and Loewenstein [31] could influence researcher decisions about accepting alcohol industry funding. For example, people who see their own research as difficult to fund, while being subject to the pressures of developing an academic career, may regard seeking industry funding as justifiable ways to advance their research. It is well established that self-reported intentions and similar attitudinal variables are weak predictors of future behaviours [32, 33]. Consequently, we have added one modification to this study paradigm, making this an unusual study; undertaking this study in a population already known to have enacted the behaviour under study. This study paradigm is thus fundamentally aetiological in nature, reminding participants of candidate influences on prior decisions to enact behaviours, in this case to seek and accept alcohol industry funding.

Employing Sah and Loewenstein’s experimental paradigm [31], our primary aim was to determine whether reminding researchers of candidate views (i.e., making them salient) would affect their self-reported receptivity to accepting alcohol industry funding now, as a measure of how they actually made this decision previously, if assumptions are made about lack of change over time. This study design also implies some capacity for evaluation of the validity of self-report [34] in comparison with an external data source. We considered entitlement a construct worth investigating and sought to develop at least one other candidate for experimental study on the basis of our knowledge of the field (see Methods for construct and intervention development). As a secondary aim, we examined whether recipients of funding for biomedical and psychosocial research differed in their openness to working with the alcohol industry.

## Methods

We employed a mixed-methods pilot study design with construct and instrument development, prior to undertaking an online survey and nested experiment. The experiment involved randomly allocating industry funded researchers to one of three groups to receive reminders about entitlement, reminders about a newly developed target for study (CoI contrarianism, see below), or no such reminders (control). As this was not a clinical trial, we pre-registered details of the study design, data management, secondary outcomes, psychometric analysis, and exploratory analysis plans along with a discussion of ethical issues raised on 2021-01-07 in the Open Science Framework (https://osf.io/jv7yz). There is one minor deviation to report. In the exploratory psychometric analyses in Additional File 4 we do not report omegas or any other reliability estimates. Although this is a developing area, the current consensus is that model-based reliability estimates should be based solely on confirmatory models (see [35] for an example). We report the factor loadings in full in Additional File 4.

### Construct and instrument development

We did a pilot study to explore perceptions of entitlement, invulnerability, and contrarianism among 25 researchers recruited from different health fields. The researchers completed the questionnaire, and provided qualitative data. CoI was prominent in the responses in a range of different ways, as it is in the literature (e.g [36]), shaping how researchers engaged with the task. We retained the entitlement construct for the experiment, dropping one item from the measure. The pilot data did not provide support for invulnerability as a candidate for further study, nor contrarianism as we’d originally conceptualised and sought to measure it. For a second construct we combined items from the pilot study and generated new ones, paying close attention to distinguishing a CoI construct from both reactance (see below) and from libertarian themes as follows:

#### Conflict of interest contrarianism

(CoI-C) reflects views that CoI is unimportant and unproblematic. This is different from being critical of CoI-related practices because one sees CoI guidance as weakly conceived or currently unhelpful. Contrarianism is related to, but distinct from, regarding attention to this subject as infringing upon the autonomy of researchers to make their own decisions. CoI-C is defined by opposition in principle to ideas concerning CoI, and rejection of their legitimacy. We hypothesised that researchers who have more contrarian views on CoI (as indicated by higher scores) would be more willing to accept industry funding and otherwise work with industry.

#### Entitlement

This refers to seeing one’s own research interests as difficult to pursue in the contexts of available funding, and the pressures of developing an academic career, which makes accepting industry funding and forming other connections with industry, reasonable ways to advance one’s research. We hypothesised that researchers with a greater sense of entitlement (as indicated by higher scores) would be more willing to accept industry funding and otherwise work with industry.

#### Outcome measurement

We generated a composite outcome measure of openness to working with the alcohol industry based on our knowledge of the area and the issues (see Additional File 1). It performed well in the pilot study, and we made no changes to it for the hypothesis testing phase of the research.

#### Additional perspectives

Following the experimental component of the survey, we asked questions on other perspectives that are critical of CoI, academic reactance, socialization, peer influences, general psychological reactance, and other issues (see Additional File 1 for all items). These questions were asked of all three groups after they completed the outcome measure.

We presented Likert scale response options (scored 1–5) for all questions. The sum of response scores to the 9 outcome measure questions (minimum possible score 9; maximum 45) made up the primary outcome measure of openness to working with industry in the experimental part of the study (with higher scores indicating more openness).

### Participants and recruitment

**Fig. 1:**
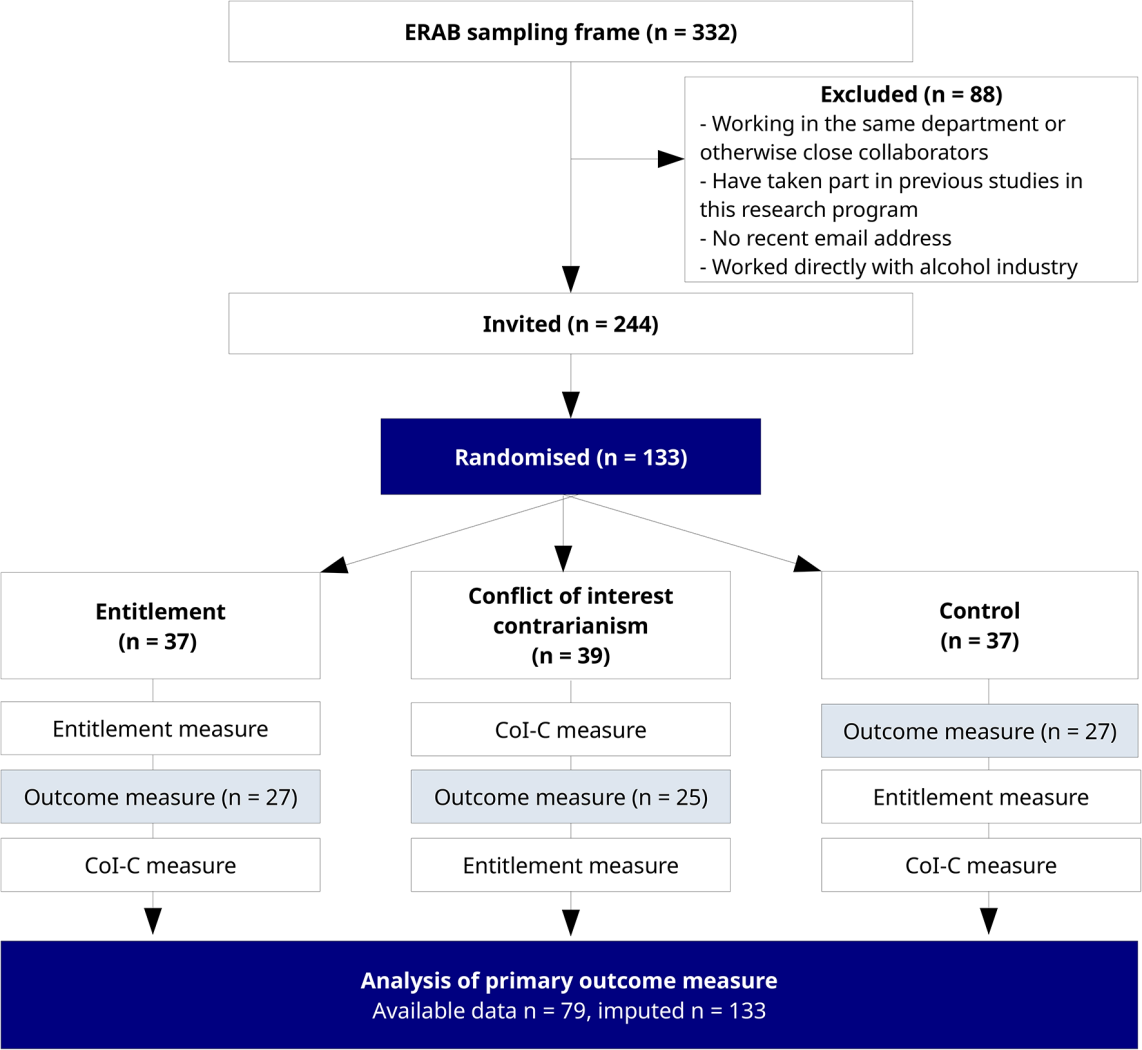
– CONSORT flow diagram

The study design required a study population who had received alcohol industry funding. The sampling frame we used was a list of researchers who had been recipients of funding provided by the European Research Advisory Board (ERAB), which was funded by the Brewers of Europe. We included all recipients listed online from inception in 2003 to 2019 (ERAB was closed in 2020). We identified a total of 332 e-mail addresses from ERAB online publications of grants awarded and filtered those as shown in Fig. 1.

There were no selection criteria other than as reported in Fig. 1. This process yielded a total of 244 e-mail addresses for survey invitations. The sample included both those who were principal investigators and co-investigators. An e-mail invitation was sent to all included researchers in January 2021. We sent three reminder e-mails inviting participation at one to two week intervals to those who had not replied, then a final e-mail to non-respondents advising them that their participation was no longer required.

### Study conditions

Researchers who clicked on a link in the invitiation email were randomized to one of three conditions and presented with an online survey consisting of 34 items (1–3 fewer due to branching if reporting no prior industry funding or being unsure). The survey took approximately 10 minutes to complete. Depending on group allocation (entitlement, CoI-C, or control), the survey was constructed as follows:

- The entitlement group received the 5 entitlement questions before the 9-item outcome measure, followed by the CoI-C and the additional perspectives questions.
- The CoI-C group received the 4 conflict of interest contrarianism questions before the 9-item outcome measure, followed by the entitlement and additional perspectives questions.
- The control group answered the 9-item outcome measure, followed by the entitlement, CoI-C and additional perspectives questions.

Delivery of the experimental conditions and measurement of outcome occurred within a single session.

### Blinding

We sent potential participants an e-mail message inviting them to consent to a study of research funding involving answering 34 questions in an online survey. Participants were thus masked to the specific aims of the research and the randomised experiment. The e-mail itself contained the information needed to participate, and clicking on the link automatically randomised participants to one of three versions of the questionnaire. After participants finished the survey we offered them debriefing, explaining that the usual process of fully informed consent would have undermined the purpose of the study. None raised concerns about the ethics of this study.

### Randomisation

We used stratified block randomisation (1:1:1 to each group) acccording to whether funding received was categorised as biomedical or psychosocial. Six researchers received both types of funding, five of which were allocated to the biomedical stratum after examination of their research interests, and one to psychosocial. Three categorized by the funder as epidemiological were allocated to the psychosocial stratum. This yielded strata of 161 and 83, respectively, for biomedical and psychosocial sample sizes. We employed randomisation with block sizes varying from 2 to 4, using the *blockrand* package (version 1.5) in R (version 4.05). All procedures were fully automated by the backend server, so that the progamming preserved the integrity of sequence generation and allocation concealment.

### Statistical methods

All analyses were intention-to-treat, using imputation when necessary in the primary and secondary analyses. Bayesian inference was used to estimate regression models [37]. Unlike the frequentist paradigm—which typically relies on null hypothesis testing to rule out the assumed scenario of no effect—the Bayesian paradigm produces a so-called posterior distribution over the effect of interest, which quantifies the compatibility of different effect estimates with the data observed. This allows the probability of an effect to be calculated directly, rather than via the probability of observed data occurring if the true effect is the null. In other words, the Bayesian paradigm does not dichotomise the evidence into statistically significant or not, rather, the posterior distribution is the result of the analysis, which then requires scientific interpretation.

We used Markov Chain Monte Carlo (MCMC) methods in *RStan* (the R interface to Stan. 2020) to produce Bayesian estimates. Given the exploratory nature of the study, we adopted wide standard normal priors for coefficients (mean = 0, standard deviation = 5) to avoid ‘over-shrinkage’ which is unduly conservative [37]. We report the posterior distribution medians and 95% compatibility intervals (defined by the 2.5% and 97.5% percentiles of the posterior distribution). We imputed missing values at the same time as we estimated regression coefficients, letting parameter samples in each of the MCMC iterations define normal distributions from which imputed values were drawn.

The primary analysis compared outcome measure scores between the three groups, regressing (linear) the outcome score on group allocation (dummy variable with 0 = control as the reference category, 1 = entitlement, 2 = CoI-C as categories) and adjusting for the stratifying variable (research area: biological vs. psychosocial). Secondary analyses estimated interactions between group allocation and scores on the entitlement and CoI-C measures, with the outcome measure.

In a sensitivity analysis we explored differences in effects between participants who did or did not report having received research funding from the alcohol industry. This was done by adding an interaction term to the primary regression model between group allocation and a dummy variable (0 = report having received, 1 = report not having received). The results revealed analytic issues that compromised the utility of this approach as a sensitivity analysis, which we discuss below.

### Sample size

We anticipated a response rate of 40% from the list of email addresses we collated, resulting in a target sample size of 98 participants. Our sample size estimates indicated that this number would not yield high precision in the effect estimates. We accepted this compromise knowing that, subject to the exclusion criteria reported in Fig. 1, we were inviting the entire known population to participate, and judged that producing a posterior distribution over effect estimates would be informative in the context of a novel, exploratory study.

## Results

Of the 244 researchers invited, 113 (46.3%) initially consented by clicking on the link in the email and were subsequently randomised. Figure 1 presents a CONSORT flowchart of particpents through the study. In response to the first email invitation, 73 participants consented by clicking on the link, though only 45 (61.6%) completed the outcome measure, and almost all the remaining participants did not respond to any questions at all. To address this issue, we confirmed the intent of the survey by replicating the information in the email at the top of the web page on which the survey was presented and included the University of York logo. We sent up to four reminder emails, to which 40 participants consented by clicking on the link. Of these, 34 (85%) completed the outcome measure, yielding a total sample size of 79 for the experiment, short of our anticipated *n* = 98 (with *n* = 76 completing the entire questionnaire), by February 2021.

**Table 1:** Number of participants by location

	Invited	Randomised (% of those invited)	Completed outcome (% of those randomised)
Germany	29	6 (20.7%)	4 (66.7%)
Italy	35	21 (60.0%)	14 (66.7%)
Netherlands	40	20 (50.0%)	13 (65.0%)
Spain	28	11 (39.3%)	11 (100%)
United Kingdom	48	29 (60.4%)	21 (72.4%)
Rest of Europe	53	23 (43.4%)	14 (60.9%)
Elsewhere	11	3 (27.3%)	2 (66.7%)
**Total**	**244**	**113 (46%)**	**79 (70%)**

Table 1 shows that levels of participation were similar across countries except Germany, where researchers were less likely to participate, and Spain, where all of the researchers who clicked on the link completed the outcome measure. The control group included 15 researchers receiving funding in the biomedical category and 12 in the psychosocial category. Corresponding numbers for the entitlement group were 17 and 10, respectively, and in the CoI-C group they were 16 and 9, respectively.

**Table 2:** Openness to working with industry outcome scores* by study group and funding stratum

	Control		Entitlement		Conflict of Interest contrarianism (CoI-C)	
	Biomedical	Psychosocial	Biomedical	Psychosocial	Biomedical	Psychosocial
Mean (sd)	30.6 (7.02)	28.5 (9.96)	31.6 (5.01)	24.9 (5.95)	33.8 (5.39)	32.0 (9.60)
Combined mean (sd)	29.7 (8.34)		29.1 (6.21)		33.2 (7.05)	

* scores can range between 9 and 45 with higher scores indicating more openness to working with industry.

In Table 2, the distribution of outcome measure scores by randomised and stratified group are presented (from the 79 complete cases; see Additional File 2 for the scores on individual outcome items). Table 3 includes estimates of effects from the linear regression models using both complete-case and imputed data, which were both adjusted for biomedical and psychosocial funding. Using complete case data, we found that there was a 94.8% probability that participants in the CoI-C group responded to be more open to working with the alcohol industry, with a point estimate of effect of 2.89 (95% CI = -0.61, 6.41). On the other hand, there was no marked evidence of the entitlement group reporting being more or less open to working with the alcohol industry. A noteworthy finding was that there was a 98.0% probability that participants with psychosocial funding reported being less open to working with the alcohol industry, with a point estimate of effect of -3.19 (95% CI = -6.21; -0.16).

Table 3 shows that results were not markedly different when imputing missing data to account for all randomised participants versus analysing only participants with complete data. Consequently, complete data only were used in further analyses.

**Table 3:** Difference in openness to work with industry outcome score* by group adjusted for funding stratum

	Complete case analyses (*n* = 79)		Imputed data analyses (*n* = 113)	
	Median^a^ 95% CI	Post. prob^b^ >/< 0	Median^a^ 95% CI	Post. prob^b^ >/< 0
Entitlement vs. Control	-0.83 (-4.27; 2.62)	68.1%	-0.83 (-3.78; 2.14)	71.1%
CoI-Contrarianism vs. Control	2.89 (-0.61; 6.41)	94.8%	2.97 (-0.04; 5.96)	97.4%
Psychosocial vs. Biomedical	-3.19 (-6.21, -0.16)	98.0%	-3.30 (-5.86; -0.72)	99.4%

^a^ The median of the posterior distribution over linear effects, with 2.5% and 97.5% percentiles representing a compatibility interval (CI).

^b^ The proportion of the posterior distribution over linear effects which is in the direction of the median.

* scores can range between 9 and 45 with higher scores indicating more openness to working with industry.

### Sensitivity and secondary analyses

Detailed results from the planned sensitivity analyses and the secondary analyses are presented in Additional File 3. These show that many participants reported not being aware ERAB was funded by the alcohol industry. The secondary findings indicated that those reporting higher scores on the entitlement and CoI-C measures also reported higher outcome scores, however, while the effects were in the anticipated direction, the precision of estimates was too low to support reliable inferences.

The planned psychometric analyses are presented in Additional File 4 and suggest that entitlement was not measured as a unidimensional construct.

On finding differences in outcome scores between the funding strata we undertook one unplanned analysis, the results of which are presented in Table 4. This post hoc examination suggests the possibility of an interaction between stratum and randomised group. The analysis shows that the findings for CoI-Contrarianism are similar in both strata, whereas for entitlement the findings are different between psychosocial and biomedical researchers, as was seen in Table 3.

**Table 4:** Post-hoc experimental contrasts in each funding category

	Median^a^ 95% CI	Posterior prob.^b^ >/< 0
**Biomedical**		
Entitlement vs. Control	0.25 (-3.69; 4.10)	55.0%
CoI-Contrarianism vs. Control	2.63 (-1.32; 6.53)	90.4%
**Psychosocial**		
Entitlement vs. Control	-2.96 (-7.99; 2.16)	87.5%
CoI-Contrarianism vs. Control	3.37 (-1.88; 8.55)	89.7%

^a^ The median of the posterior distribution over linear effects, with 2.5% and 97.5% percentiles representing a compatibility interval (CI).

^b^ The proportion of the posterior distribution over linear effects which is in the direction of the median.

## Discussion

This was a novel investigation of research funding decision making among alcohol industry-funded researchers, and it should be regarded as exploratory. As such, we underline the need for caution in interpretation, also in view of study limitations (see below). The findings suggest the possibility of an effect of CoI-Contrarianism on openness to working with the alcohol industry, as reminding industry funded researchers that they were opposed to attention to CoI led them to self-report being more receptive to industry funding. This may mean that this was an important factor in decisions previously made to seek alcohol industry funding. An important caveat to all findings is that participants’ decision-making processes in the present study may be different to the decisions they actually made to seek industry funding, which for many was some time ago. In addition, the small sample size prohibits more precise estimation of effects, which means that there is still uncertainty regarding the magnitude of the effect of CoI-Contrarianism on openness to working with the alcohol industry.

The nature of the study population and the limitations of the study design warrant careful consideration as there are risks that processes of reverse causation are involved, i.e. having industry funding and conflicts of interest produce contrarian views on the subject, not the other way. The findings of this study, we suggest, are confined to what may only be one part of the causal chain; the study population having been defined by prior receipt of industry funding. It is likely that there are complex inter-relationships to be uncovered, perhaps using tools such as mediational analyses. Further and larger samples of recipients of alcohol industry and other commercial sources of research funding are available for such studies. There may also be value in an experimental study designed to rule out reverse causation in which researchers who have not had industry funding or other CoI, are randomly assigned to a decision-making intervention, with prospective observation of effects on objectively measurable outcomes. Such designs may be best employed for early career study populations at recruitment and could rely on published data for outcome evaluation in order to minimise selection and information biases. Attrition is a threat to valid inference in the present study given the numbers targeted and providing data, though note this was not differential between randomised arms.

These observations on the limitations of the present study reinforce our earlier awareness of the lack of literature on researcher decision-making. It has long been known that survey and interview studies have shown that researchers with industry funding are more likely to be favourable towards interactions with industry [38]. Low levels of awareness among researchers of the risks of industry funding is one target for change [36], as are exaggerated views held by clinicians and researchers about their ability to manage risks relating to industry funding [7]. The value of this study is thus both explanatory and methodological, and it contributes to the wider under-developed literature.

The measurement and construct validity of the outcome measure require careful consideration. The measure was designed for the purposes of the present study, and apart from our pilot work where it performed satisfactorily and was not amended, it has not previously been validated. The psychometric analyses available in Additional File 4 will be useful to researchers seeking to develop items and study this area more broadly. Entitlement did not perform well as a measure of a unidimensional construct in this study, and that has implications for the interpretation of the experimental findings. Accordingly, we conclude that the construct itself requires rethinking.

We interpret CoI-Contrarianism to have performed reasonably well in exploratory analyses, albeit with room for improvement. Note that in this study the measure comprised only four items, and the development of additional items is a promising direction for further study. The confirmatory analyses showed that the psychometric performance of all three of our measures could have been better (even if correcting for brevity of the instruments), but that our outcome measure reliably assessed individual differences.

Our planned sensitivity analysis failed due to the high numbers of researchers reporting that they had not received any alcohol industry funding, so we could not exclude their data as planned (see Additional File 3). On debriefing we asked participants the question “Did you know that ERAB was entirely funded by the alcohol industry?” Presented with binary yes/no response categories, 9 of the 40 who responded stated no, indicating they were unaware of this. This in itself is a noteworthy finding of this study, precluding any effort to study the validity of self-report of alcohol industry funding. The transparency of industry research funding requires careful consideration.

Recent evidence shows that alcohol industry involvement in science is deeper and wider than previously understood [39], with similarities to other industries [40–42]. The closure of the alcohol industry funded research organisations indicates a shift in strategy for reasons which are unclear. Contrarianism in respect of the emerging norms on COI, looks if anything more important to study now, not only for alcohol, but also more widely. Given the modest nature of this study, albeit in seeking to expand a meagre literature, it is appropriate to draw out some possible implications of study findings, whilst being cautious in so doing. If further studies replicate the importance of CoI-Contrarianism, then this may suggest such ideas should be addressed in the education and training of alcohol and health researchers more broadly. Study findings indicate this may be particularly useful for biomedical researchers, though we suggest there may be value for psychosocial researchers too. There is now substantial evidence spanning many fields of adverse impacts of industry funding on the conception, design, conduct and dissemination of research [36, 42–48], providing a firm foundation for evidence-based research training. More broadly, there is a need to develop rules and norms around COI, and change incentives structures, so that the threats that industry funding poses to the integrity of research are more widely understood.

Our conclusions are: (1) to suggest that entitlement needs to be conceptualised and measured differently than in relation to pharmaceutical gifting as a possible reason for openness to industry funding; (2) CoI-Contrarianism is important to further study; and (3) that it is possible to study researcher decision-making using quantitative methods such as were employed here. We therefore suggest that the major contribution of this study is to draw attention to the area of how researchers make decisions about industry funding in areas such as alcohol, which in turn shapes the questions scientists ask, and the answers they produce.

### Electronic supplementary material

Below is the link to the electronic supplementary material.

Supplementary Material 1

Supplementary Material 2

Supplementary Material 3

Supplementary Material 4

## Data Availability

The datasets generated and/or analysed during the current study are not publicly available due to the sensitive nature of the data as outlined in the OSF published protocol and in line with ethical approval. The first author may be contacted to further discuss data availability issues.

## Abbreviations

COI: Conflict of interest
CoI: Conflict of interest contrarianism
ERAB: European Research Advisory Board
MCMC: Markov Chain Monte Carlo
