# Supplementary material for: Randomised study of the effects of sense of entitlement and conflict of interest contrarianism on researcher decision-making to work with the alcohol industry
Source: BMC Public Health. 2024 Jun 24;24:1680. doi: 10.1186/s12889-024-18961-5 (PMC11197317; doi:10.1186/s12889-024-18961-5)
Supplement: Supplementary file 1 — Supplementary Material 1 [file 12889_2024_18961_MOESM1_ESM.docx]

**This additional file contains material accompanying McCambridge, J., Kypri, K., Boehnke, J.R., Bero, L. & Bendtsen, M. Randomised study of the effects of making salient sense of entitlement and conflict of interest contrarianism on researcher willingness to work with the alcohol industry**

**Additional File 1: Questionnaire items**

**Questionnaire items**

This appendix presents the questionnaire items as used in the study. Responses to all items were captured with five response categories ("1 = Strongly disagree", "2 = Disagree", "3 = Neutral", “4 = Agree". to "5 = Strongly agree").

*Five* ***entitlement*** *questions*

(E1) "It has been hard to establish a career in research"

(E2) "It has been difficult to get research grants from prestigious funders”

(E3) "Academic jobs are not well paid"

(E4) "Research grants given are not usually enough to do the study properly"

(E5) "I have regularly worked more than 50 hours per week"

*Four* ***conflict of interest contrarianism*** *questions*

(C1) "Too much is made of funding declarations "

(C2) "I am well placed to assess conflict of interest"

(C3) "Issues to do with industry funding are not very relevant to my area"

(C4) “Conflict of interest is not a problem”

*Nine questions comprising the* ***outcome measure*** (preceded by an instruction that in these questions industry refers to alcohol industry only)

(O1) "I think it is okay to give a talk at a conference which is industry sponsored"

(O2) "I think it is okay to give a talk at an industry organised event"

(O3) "I think it is okay to accept a fee from industry for a talk"

(O4) "I think it is okay to accept expenses from an industry source"

(O5) "I think it is okay to undertake research directly funded by industry"

(O6) "I think it is okay to undertake research funded by organisations that are funded in turn by industry"

(O7) "I think it is okay to undertake peer review for industry funded organisations"

(O8) "I think it is okay to meet with industry employees to discuss my research"

(O9) "I think it is okay to work with research colleagues who undertake research funded by industry"

*Four* ***validation and current status questions*** *(not more than three of which were asked of any participant)*

“Have you ever received research funding from any type of company or industry funded organisation?” (yes/no/unsure)

“From which industry sector(s) have you received funding?” (pharma, alcohol, food, tobacco, gambling, other (which), select all that apply)

“Would you do so again?” (if any selected)

“Why are you unsure?” (This question is shown only if previously answered ‘not sure’ and in place of the previous two questions)

***Additional items*** *exploring additional or related aspects of the three main constructs for exploratory observational study (13 overall)*

1. Critical of conflict of interest

(S_A1) "Conflict of interest issues are much more complex than are usually presented"

(S_A2) "Disclosure statements on research papers are not very helpful"

1. Academic reactance

(S_B1) "Academic freedom gives me the right to make my own decisions about my work"

(S_B2) "University bureaucrats should not interfere with my research"

1. Socialization

(S_C1) "My training prepared me well for decision-making on funding in research"

(S_C2) “My mentor was relaxed about industry funding”

1. Peer factors

(S_D1) "I trust the people I know who receive industry funding"

(S_D2) "My work has not been valued as much as it could be by my peers"

1. General psychological reactance

(S_E1) “Advice and recommendations induce me to do just the opposite”

(S_E2) “I resist the attempts of others to influence me”

1. Other

(S_F1) “I assume that my co-authors do not have any conflicts of interest unless they tell me”

(S_F2) “Conflicts of interest are different in my research area”

(S_F3) “We do not need better guidance on thinking through conflict of interest issues”
