# Supplementary material for: Randomised study of the effects of sense of entitlement and conflict of interest contrarianism on researcher decision-making to work with the alcohol industry
Source: BMC Public Health. 2024 Jun 24;24:1680. doi: 10.1186/s12889-024-18961-5 (PMC11197317; doi:10.1186/s12889-024-18961-5)
Supplement: Supplementary file 2 — Supplementary Material 2 [file 12889_2024_18961_MOESM2_ESM.docx]

**This additional file contains material accompanying McCambridge, J., Kypri, K., Boehnke, J.R., Bero, L. & Bendtsen, M. Randomised study of the effects of making salient sense of entitlement and conflict of interest contrarianism on researcher willingness to work with the alcohol industry**

**Additional File 2: Distribution of scores on individual outcome measure items**

The tables in this section describe the distribution of response scores to the 9 outcome measure questions by strata and group (complete cases, n = 79). Mean and standard deviation (sd) are shown both for each stratum and combined.

| **O1** - I think it is okay to give a talk at a conference which is industry sponsored | | | | | | |
| --- | --- | --- | --- | --- | --- | --- |
| Stratum | Biomedical | Psychosocial | Biomedical | Psychosocial | Biomedical | Psychosocial |
| Group | Control | Control | Entitlement | Entitlement | CoI-C | CoI-C |
| Mean (sd) | 3.60 (0.83) | 3.25 (1.42) | 3.53 (0.87) | 2.70 (0.82) | 3.81 (0.91) | 3.56 (1.24) |
| Combined mean (sd) | 3.44 (1.12) | | 3.22 (0.93) | | 3.72 (1.02) | |

| **O2** - I think it is okay to give a talk at an industry organised event | | | | | | |
| --- | --- | --- | --- | --- | --- | --- |
| Stratum | Biomedical | Psychosocial | Biomedical | Psychosocial | Biomedical | Psychosocial |
| Group | Control | Control | Entitlement | Entitlement | CoI-C | CoI-C |
| Mean (sd) | 3.13 (1.06) | 3.08 (1.51) | 3.65 (0.61) | 2.80 (0.79) | 3.44 (0.89) | 3.44 (1.01) |
| Combined mean (sd) | 3.11 (1.25) | | 3.33 (0.78) | | 3.44 (0.92) | |

| **O3** - I think it is okay to accept a fee from industry for a talk | | | | | | |
| --- | --- | --- | --- | --- | --- | --- |
| Stratum | Biomedical | Psychosocial | Biomedical | Psychosocial | Biomedical | Psychosocial |
| Group | Control | Control | Entitlement | Entitlement | CoI-C | CoI-C |
| Mean (sd) | 2.47 (1.13) | 2.83 (1.59) | 2.94 (1.03) | 1.30 (0.48) | 3.38 (1.09) | 3.22 (1.48) |
| Combined mean (sd) | 2.63 (1.33) | | 2.33 (1.18) | | 3.32 (1.22) | |

| **O4** - I think it is okay to accept expenses from an industry source | | | | | | |
| --- | --- | --- | --- | --- | --- | --- |
| Stratum | Biomedical | Psychosocial | Biomedical | Psychosocial | Biomedical | Psychosocial |
| Group | Control | Control | Entitlement | Entitlement | CoI-C | CoI-C |
| Mean (sd) | 3.20 (1.26) | 2.67 (1.44) | 3.35 (0.86) | 1.90 (0.88) | 3.81 (0.83) | 3.22 (1.39) |
| Combined mean (sd) | 2.96 (1.34) | | 2.81 (1.11) | | 3.60 (1.08) | |

| **O5** - I think it is okay to undertake research directly funded by industry | | | | | | |
| --- | --- | --- | --- | --- | --- | --- |
| Stratum | Biomedical | Psychosocial | Biomedical | Psychosocial | Biomedical | Psychosocial |
| Group | Control | Control | Entitlement | Entitlement | CoI-C | CoI-C |
| Mean (sd) | 3.33 (1.35) | 2.50 (1.38) | 3.29 (0.92) | 2.60 (1.17) | 3.75 (1.06) | 3.11 (1.45) |
| Combined mean (sd) | 2.96 (1.40) | | 3.04 (1.06) | | 3.52 (1.23) | |

| **O6** - I think it is okay to undertake research funded by organisations that are funded in turn by industry | | | | | | |
| --- | --- | --- | --- | --- | --- | --- |
| Stratum | Biomedical | Psychosocial | Biomedical | Psychosocial | Biomedical | Psychosocial |
| Group | Control | Control | Entitlement | Entitlement | CoI-C | CoI-C |
| Mean (sd) | 3.80 (0.86) | 2.92 (1.38) | 3.53 (0.80) | 3.10 (1.10) | 4.06 (0.57) | 3.67 (1.32) |
| Combined mean (sd) | 3.41 (1.19) | | 3.37 (0.93) | | 3.92 (0.91) | |

| **O7** - I think it is okay to undertake peer review for industry funded organisations | | | | | | |
| --- | --- | --- | --- | --- | --- | --- |
| Stratum | Biomedical | Psychosocial | Biomedical | Psychosocial | Biomedical | Psychosocial |
| Group | Control | Control | Entitlement | Entitlement | CoI-C | CoI-C |
| Mean (sd) | 3.47 (0.99) | 3.67 (0.78) | 3.53 (0.80) | 3.00 (1.15) | 3.75 (0.86) | 3.67 (1.32) |
| Combined mean (sd) | 3.56 (0.89) | | 3.33 (0.96) | | 3.72 (1.02) | |

| **O8** - I think it is okay to meet with industry employees to discuss my research | | | | | | |
| --- | --- | --- | --- | --- | --- | --- |
| Stratum | Biomedical | Psychosocial | Biomedical | Psychosocial | Biomedical | Psychosocial |
| Group | Control | Control | Entitlement | Entitlement | CoI-C | CoI-C |
| Mean (sd) | 3.73 (0.96) | 3.92 (1.08) | 3.94 (0.56) | 3.80 (1.14) | 3.69 (0.95) | 4.11 (0.93) |
| Combined mean (sd) | 3.81 (1.00) | | 3.89 (0.80) | | 3.84 (0.94) | |

| **O9** - I think it is okay to work with research colleagues who undertake research funded by industry | | | | | | |
| --- | --- | --- | --- | --- | --- | --- |
| Stratum | Biomedical | Psychosocial | Biomedical | Psychosocial | Biomedical | Psychosocial |
| Group | Control | Control | Entitlement | Entitlement | CoI-C | CoI-C |
| Mean (sd) | 3.87 (0.74) | 3.67 (0.89) | 3.82 (0.64) | 3.70 (0.67) | 4.12 (0.50) | 4.00 (0.87) |
| Combined mean (sd) | 3.78 (0.80) | | 3.78 (0.64) | | 4.08 (0.64) | |
