# Supplementary material for: Randomised study of the effects of sense of entitlement and conflict of interest contrarianism on researcher decision-making to work with the alcohol industry
Source: BMC Public Health. 2024 Jun 24;24:1680. doi: 10.1186/s12889-024-18961-5 (PMC11197317; doi:10.1186/s12889-024-18961-5)
Supplement: Supplementary file 3 — Supplementary Material 3 [file 12889_2024_18961_MOESM3_ESM.docx]

**This additional file contains material accompanying McCambridge, J., Kypri, K., Boehnke, J.R., Bero, L. & Bendtsen, M. Randomised study of the effects of making salient sense of entitlement and conflict of interest contrarianism on researcher willingness to work with the alcohol industry**

**Additional File 3: Sensitivity and Secondary Analyses**

*Sensitivity Analysis 1*

In sensitivity analysis 1, both reporting having received alcohol industry funding as well as being unsure was considered *self-reporting positive*, while reporting not having received alcohol industry funding in the past was considered *self-reporting negative*. In sensitivity analysis 2, being unsure was moved from *self-reporting positive* to *self-reporting negative*.

|  | **Median^a^ 95% CI** | **Posterior prob.^b^  >/< 0** |
| --- | --- | --- |
| **Self-report negative (n = 42)** | | |
| Entitlement vs. Control | -1.59  (-5.65; 2.47) | 77.9% |
| CoI-Contrarianism vs. Control | 2.67 (-1.68; 7.04) | 88.7% |
| **Self-report positive (n = 34)** | | |
| Entitlement vs. Control | -2.39 (-7.66; 2.99) | 81.1% |
| CoI-Contrarianism vs. Control | 0.61 (-4.59; 5.84) | 59.1% |
| Positive vs. Negative | 4.05 (-0.46; 8.48) | 96.1% |
| **Common** | | |
| Psychosocial vs. Biomedical | -3.11 (-6.21; -0.02) | 97.6% |
| ^a^ The median of the posterior distribution over linear effects, with 2.5% and 97.5% percentiles representing a compatibility interval (CI).  ^b^ The proportion of the posterior distribution over linear effects which is in the direction of the median. | | |

*Sensitivity Analysis 2*

In sensitivity analysis 2, being unsure was moved from *self-reporting positive* to *self-reporting negative*.

|  | **Median^a^ 95% CI** | **Posterior prob.^b^  >/< 0** |
| --- | --- | --- |
| **Self-report negative (n = 44)** | | |
| Entitlement vs. Control | -1.62 (-5.61; 2.39) | 78.6% |
| CoI-Contrarianism vs. Control | 2.94 (-1.32; 7.16) | 91.3% |
| **Self-report positive (n = 32)** | | |
| Entitlement vs. Control | -2.09 (-7.49; 3.36) | 77.9% |
| CoI-Contrarianism vs. Control | 0.50 (-4.47; 5.78) | 57.3% |
| Positive vs. Negative | 4.01 (-0.50; 8.45) | 96.0% |
| **Common** | | |
| Psychosocial vs. Biomedical | -3.13 (-6.21; -0.02) | 97.6% |
| ^a^ The median of the posterior distribution over linear effects, with 2.5% and 97.5% percentiles representing a compatibility interval (CI).  ^b^ The proportion of the posterior distribution over linear effects which is in the direction of the median. | | |

*Secondary Analysis 1 - Entitlement scores and outcome*

Regression of outcome scores on entitlement vs control with an interaction with the summed score of the entitlement questions, adjusted for research area (stratification variable).

|  | **Median^a^ 95% CI** | **Posterior prob.^b^  >/< 0** |
| --- | --- | --- |
| **Control group (n = 37)** | | |
| +1 entitlement measure | 0.14 (-0.30; 0.57) | 73.7% |
| **Entitlement group (n = 37)** | | |
| +1 entitlement measure | 0.23 (-0.37; 0.83) | 77.7% |
| Entitlement vs. Control | -2.32 (-11.35; 6.70) | 69.3% |
| **Common** | | |
| Psychosocial vs. Biomedical | -3.73 (-7.42; -0.05) | 97.6% |
| ^a^ The median of the posterior distribution over linear effects, with 2.5% and 97.5% percentiles representing a compatibility interval (CI).  ^b^ The proportion of the posterior distribution over linear effects which is in the direction of the median. | | |

*Secondary Analysis 2 - Conflict of interest contrarianism scores and outcome*

Regression of outcome on conflict of interest contrarianism vs control with an interaction with the summed score of the conflict of interest contrarianism questions, adjusted for research area (stratification variable).

|  | **Median^a^ 95% CI** | **Posterior prob.^b^  >/< 0** |
| --- | --- | --- |
| **Control group (n = 37)** | | |
| +1 contrarian measure | 0.18 (-0.48; 0.83) | 70.9% |
| **Conflict of interest contrarianism group (n = 39)** | | |
| +1 contrarian measure | 0.17 (-0.65; 1.00) | 66.1% |
| CoI-Contrarianism vs. Control | 2.92 (-5.37; 11.1) | 75.4% |
| **Common** | | |
| Psychosocial vs. Biomedical | -1.89 (-5.96; 2.26) | 81.8% |
| ^a^ The median of the posterior distribution over linear effects, with 2.5% and 97.5% percentiles representing a compatibility interval (CI).  ^b^ The proportion of the posterior distribution over linear effects which is in the direction of the median. | | |

*Secondary Analysis 3 and 4*

We regressed outcome scores on entitlement vs control with interactions with the score of each separate entitlement question, adjusted for research area (stratification variable). Similarly, we regressed outcome scores on contrarianism vs control with interactions with the score of each separate contrarianism question, adjusted for research area (stratification variable). These two models were estimated using shrinkage priors and none of the covariate estimates “escaped” the shrinkage, thus no marked association between individual questions and group allocation on the outcome score was observed.
