# Supplementary material for: Randomised study of the effects of sense of entitlement and conflict of interest contrarianism on researcher decision-making to work with the alcohol industry
Source: BMC Public Health. 2024 Jun 24;24:1680. doi: 10.1186/s12889-024-18961-5 (PMC11197317; doi:10.1186/s12889-024-18961-5)
Supplement: Supplementary file 4 — Supplementary Material 4 [file 12889_2024_18961_MOESM4_ESM.docx]

**This additional file contains material accompanying McCambridge, J., Kypri, K., Boehnke, J.R., Bero, L. & Bendtsen, M. Randomised study of the effects of making salient sense of entitlement and conflict of interest contrarianism on researcher willingness to work with the alcohol industry**

**Additional File 4: Psychometric Analyses**

*Confirmatory factor analyses*

We fitted unidimensional confirmatory factor analyses to the entitlement, conflict of interest (coi) contrarianism, and outcome measures to evaluate how well the item responses can be represented by the single scores used in the analyses (analyses conducted with lavaan, Rosseel, 2012; Revelle, 2022). This step was undertaken to evaluate how well the item responses can be represented by the single scores used in the analyses. We calculated coefficient *ω* based on the factor loadings from these models as a measure of reliability (McDonald, 1999). Supplementary Material 4 Table 1 presents the resulting fit statistics and reliability estimates, and Table 2 the estimated and standardized loadings for each model. None of the three psychometric measures performed well on all criteria for a reflective measurement instrument. The five items measuring entitlement cannot be assumed to be indicators of the same latent construct: all model fit statistics failed minimum standards and while the items E1, E2, and E4 showed acceptable to excellent loadings, the other two loaded low or virtually not at all on that dimension. This is likely due to the fact that the items seem to assess more practical as well as more prestige-related sources of Entitlement (see discussion of these items in the exploratory factor analyses below). The four coi contrarianism items showed overall acceptable fit statistics, but item C2 did not load on the extracted factor. The reliability estimates for both of these scales would be considered low, even for between-group comparisons and with a short polytomous measure.

The fit for a unidimensional model for the nine outcome items was not great, but all items showed substantial factor loadings and the reliability estimate indicated that a substantial amount of variance observed in the items could be related to the single extracted factor. This is a likely result in cases where not all empirically relevant inter-relationships between items are fully captured by this single factor. Since a reliability estimate depends on the number of items used, Supplementary Material 4 Table 1 also presents estimates corrected to the same length (nine items; Spearman-Brown Prophecy Formula), which further illustrates that the outcome performed well by the sole standard of reliability; coi contrarianism is measured potentially acceptable for group comparisons; but the measure for entitlement does not meet usual expectations.

*Additional File 4 Table 1. Model fit statistics and reliability estimates for the unidimensional confirmatory factor analyses of the three implemented psychometric measures.*

|  | Entitlement  (5 items; N=78) | Conflict of Interest Contrarianism  (4 items; N=77) | Outcome  (9 items; N=79) |
| --- | --- | --- | --- |
| *χ*² Test | *χ*²*_df_*_=5_ = 18.65; *p* = .002 | *χ*²*_df_*_=2_ = 2.69; *p* = .26 | *χ*²*_df_*_=27_ = 67.91; *p* < .001 |
| CFI / TLI | .75 / .50 | .97 / .91 | .91 / .88 |
| RMSEA [90% CI] | .19 [.10; .28] | .07 [.00, .25] | .14 [.09; .18] |
| Reliability (*ω*) | .52 | .59 | .92 |
| Spearman-Brown Prophecy corrected  (9 items) | .66 | .76 | -NA- |

*Note*. CFI Comparative Fit Index; TLI Tucker Lewis Index; RMSEA Root Mean Square Error of Approximation; CI Confidence Interval; *ω* Coefficient Omega; *df* Degrees of Freedom.

*Additional File 4 Table 2. Factor loading estimates (incl. standardized results) from the unidimensional confirmatory factor analyses of the three implemented psychometric measures.*

| Outcome | | |  | Entitlement | | |  | CoI-C | | |
| --- | --- | --- | --- | --- | --- | --- | --- | --- | --- | --- |
|  | Estimate (SE) | Std |  |  | Estimate (SE) | Std |  |  | Estimate (SE) | Std |
| O1 | 1.00 | 0.91 |  | E1 | 1.00 | 0.78 |  | C1 | 1.00 | 0.54 |
| O2 | 0.87 (0.09) | 0.82 |  | E2 | 0.81 (0.24) | 0.73 |  | C2 | 0.24 (0.22) | 0.16 |
| O3 | 1.02 (0.12) | 0.74 |  | E3 | 0.37 (0.17) | 0.30 |  | C3 | 0.99 (0.38) | 0.45 |
| O4 | 1.05 (0.11) | 0.81 |  | E4 | 0.44 (0.16) | 0.40 |  | C4 | 1.68 (0.73) | 0.74 |
| O5 | 1.02 (0.12) | 0.77 |  | E5 | -0.16 (0.19) | -0.11 |  |  |  |  |
| O6 | 0.90 (0.09) | 0.82 |  |  |  |  |  |  |  |  |
| O7 | 0.71 (0.10) | 0.69 |  |  |  |  |  |  |  |  |
| O8 | 0.50 (0.10) | 0.52 |  |  |  |  |  |  |  |  |
| O9 | 0.48 (0.07) | 0.64 |  |  |  |  |  |  |  |  |

*Note. O1-O9 Outcome items, E1-E5 Entitlement items, C1-C4 coi contrarianism (COI-C) items, see Supplementary Material 1 for item content; SE Standard error; Std Standardized loading.*

*Exploratory factor analyses*

Exploratory factor analyses were used to investigate interrelationships between the items measuring entitlement, coi contrarianism, and the 13 additional newly developed items that were presented to all participants. The aim was to explore how the 13 new items related to the entitlement and coi contrarianism items, and to evaluate whether they were addressing potentially similar constructs, which was largely negated.

The parallel analysis based on the raw inter-item correlations suggested that six factors extract relevant variation compared to random data with similar distributions; and five factors after partialling out covariation associated with the experimental condition. The factors were not strongly correlated (raw data: -.15 ≤ r ≤ .19; partialled out: -.17 ≤ r ≤ .17). The solutions were very similar, please see Supplementary Material 4 Table 3 and Table 4, assuming that λ > |.30| indicate potentially relevant loadings. Both solutions showed a factor on which the three coi contrarianism items loaded, indicating that too much is made of funding declarations, that issues with industry funding are not relevant, and coi is not a problem. The item "Disclosure statements on research papers are not very helpful" (S_A2) also showed a loading on this factor, and after partialling out, a small loading was also observed for "Research grants given are not usually enough to do the study properly" (E4). This factor seems to have worked as intended by the original development and future research could develop further content in this direction. The cross-loading with E4 suggests that clarity in formulation is required so that a contrarian attitude towards coi is not mixed with other motivating factors, which especially in cross-sectional research may have spurious correlations due to their joint development (e.g., experienced or perceived hardship leads to entitlement, but also feeling more hindered/threatened by conflict of interests).

Both solutions showed a second factor dominated by the first two entitlement items, covering difficulties in establishing a career in research (E1) and getting research grants from prestigious funders (E2). Additionally, responses to these two also correlated with the attitude that coi issues are much more complex than are usually presented (S_A1), and after partialling out the experimental conditions, that researchers perceive that their work has not been valued as much as it could be by their peers (S_D2). The statements loading on this factor seem to focus aspects of career and prestige and their related entitlement.

Both solutions showed a third factor dominated by the final three entitlement items focusing on more practical aspects of (perceived) hardship: that academic jobs are not well paid, that grants are usually not enough to perform a study properly (see also discussion of the first factor above), and that they have worked regularly more than 50hrs. The additional content loading on this factor was that one assumes that co-authors do not have any coi unless they declare them (S_F1), and that there is no need for more guidance to think coi through (S_F3). Both loaded in the opposite direction to the entitlement items on this factor, and after partialling out the experimental conditions, the coi contrarianism statement that coi is not a problem (C4) loads also in the opposite direction to the entitlement items. While this factor captures entitlement due to perceived hardship, the wider response behaviour of the participants shows again as for the first factor that it may be difficult to separate between perceived hardships and the unimportance and/or role of coi as obstacles.

The fourth and fifth factors consisted mainly of additionally developed items and capture more general aspects of contrarian attitudes. The fourth factor was described by items focusing on academic freedom, supporting the right to make one's own decisions, that university bureaucrats should not interfere, and that coi are different in the particular research area. The fifth factor mixed the view of coi issues being much more complex than are usually presented with opinions re-asserting autonomy, such as that academic freedom gives me the right to make my own decisions about my work, and even active resistance in such statements, such as advice and recommendations induce me to do just the opposite, and the opinion that on resists attempts of others to influence one (strongest loading). Participants with higher scores on this component would also see themselves as less well-placed to assess coi. These factors point out clearly that in the conceptualization and measurement of especially conflicts of interest contrarianism, it is important not to tap too much into general tendencies to seek independence and autonomy, but to focus on the specific issue. That these two factors barely at all related to our original items for the two core concepts of coi contrarianism and entitlement shows that these different aspects can likely be separated.

In the raw correlation matrix, a sixth factor was found that consisted of items indicating that one's mentor was relaxed about industry funding and that participants trust people who receive industry funding; and the item that one's work has not been valued as much as it could be by one's peers shows an opposite loading on this factor. This more social aspect does not show up anymore after controlling for the experimental conditions and the third of these items is rather allocated to the second factor discussed above. The item "My training prepared me well for decision-making on funding in research" (S_C1) does not show a substantial loading on any of the extracted factors in either solution; and after partialling experimental condition out, the items "My mentor was relaxed about industry funding" and "I trust the people I know who receive industry funding" are not related to any of the extracted factors either.

*Additional File 4 Table 3. Factor loadings from an exploratory factor analysis (solution with six factors; λ > |.30| in bold face).*

|  | **PA1** | **PA2** | **PA3** | **PA4** | **PA5** | **PA6** |
| --- | --- | --- | --- | --- | --- | --- |
| E1 | -0.128 | **0.661** | 0.139 | 0.067 | -0.051 | 0.065 |
| E2 | 0.075 | **0.789** | -0.107 | 0.020 | 0.001 | 0.000 |
| E3 | 0.223 | 0.037 | **0.631** | 0.196 | 0.113 | -0.050 |
| E4 | 0.283 | 0.277 | **0.365** | 0.176 | -0.091 | 0.046 |
| E5 | 0.015 | -0.263 | **0.434** | 0.014 | -0.142 | 0.086 |
| C1 | **0.592** | 0.143 | 0.057 | 0.113 | -0.191 | 0.235 |
| C2 | 0.094 | 0.032 | 0.065 | 0.273 | -0.304 | -0.112 |
| C3 | **0.597** | -0.243 | 0.017 | -0.128 | 0.116 | -0.060 |
| C4 | **0.571** | -0.055 | -0.283 | 0.208 | -0.092 | 0.039 |
| S_A1 | -0.049 | **0.377** | 0.181 | -0.287 | **0.331** | -0.043 |
| S_A2 | **0.682** | 0.081 | 0.107 | -0.150 | 0.114 | -0.086 |
| S_B1 | -0.132 | -0.086 | 0.020 | **0.337** | **0.366** | 0.187 |
| S_B2 | -0.048 | 0.018 | 0.035 | **0.555** | 0.251 | 0.262 |
| S_C1 | 0.053 | -0.200 | -0.176 | 0.094 | 0.143 | 0.258 |
| S_C2 | -0.132 | -0.003 | -0.043 | 0.069 | 0.022 | **0.465** |
| S_D1 | 0.046 | 0.139 | -0.068 | -0.118 | -0.018 | **0.600** |
| S_D2 | -0.072 | 0.285 | 0.010 | 0.230 | 0.031 | **-0.412** |
| S_E1 | 0.233 | 0.139 | -0.239 | 0.029 | **0.383** | -0.038 |
| S_E2 | 0.024 | -0.037 | -0.005 | 0.043 | **0.711** | -0.021 |
| S_F1 | -0.044 | -0.008 | **-0.357** | 0.159 | 0.076 | -0.009 |
| S_F2 | -0.010 | 0.095 | -0.036 | **0.728** | -0.045 | -0.144 |
| S_F3 | 0.161 | 0.009 | **-0.677** | 0.170 | 0.057 | 0.087 |

*Additional File 4 Table 4. Factor loadings from an exploratory factor analysis after partialling out covariation associated with the experimental condition (solution with five factors; λ > |.30| in bold face).*

|  | **PA1** | **PA2** | **PA3** | **PA4** | **PA5** |
| --- | --- | --- | --- | --- | --- |
| E1 | -0.083 | **0.627** | -0.108 | 0.055 | -0.008 |
| E2 | 0.091 | **0.740** | 0.104 | 0.001 | 0.015 |
| E3 | 0.243 | 0.111 | **-0.553** | 0.187 | 0.089 |
| E4 | **0.301** | 0.283 | **-0.330** | 0.218 | -0.118 |
| E5 | 0.056 | -0.268 | **-0.434** | 0.068 | -0.142 |
| C1 | **0.666** | 0.068 | -0.018 | 0.126 | -0.186 |
| C2 | 0.075 | 0.172 | -0.020 | 0.090 | **-0.328** |
| C3 | **0.579** | -0.205 | 0.057 | -0.158 | 0.152 |
| C4 | **0.515** | -0.007 | **0.369** | 0.141 | -0.129 |
| S_A1 | -0.001 | **0.339** | -0.230 | -0.242 | **0.401** |
| S_A2 | **0.662** | 0.064 | -0.088 | -0.142 | 0.112 |
| S_B1 | -0.082 | -0.095 | 0.002 | **0.439** | **0.315** |
| S_B2 | 0.001 | -0.022 | -0.008 | **0.713** | 0.155 |
| S_C1 | 0.148 | -0.229 | 0.219 | 0.097 | 0.202 |
| S_C2 | 0.022 | -0.172 | 0.002 | 0.232 | 0.039 |
| S_D1 | 0.215 | -0.044 | 0.054 | 0.044 | 0.077 |
| S_D2 | -0.160 | **0.472** | 0.023 | 0.002 | -0.008 |
| S_E1 | 0.195 | 0.122 | 0.238 | 0.068 | **0.336** |
| S_E2 | -0.024 | -0.008 | 0.049 | 0.165 | **0.701** |
| S_F1 | -0.080 | 0.013 | **0.365** | 0.124 | 0.038 |
| S_F2 | -0.045 | 0.243 | 0.091 | **0.539** | -0.163 |
| S_F3 | 0.116 | 0.032 | **0.739** | 0.100 | 0.040 |

**References**

McDonald, R. P. (1999). Test theory: A unified treatment. Mahwah, NJ, Lawrence Erlbaum Associates.

Revelle W (2022). psych: Procedures for Psychological, Psychometric, and Personality Research. Northwestern University, Evanston, Illinois. R package version 2.2.3, https://CRAN.R-project.org/package=psych.

Rosseel, Y. (2012). "lavaan: An R package for Structural Equation Modeling." Journal of Statistical Software 48(2).

**Supplementary Material 5: Post-hoc experimental contrasts in each funding category**

|  | **Median^a^ 95% CI** | **Posterior prob.^b^  >/< 0** |
| --- | --- | --- |
| **Biomedical** | | |
| Entitlement vs. Control | 0.25 (-3.69; 4.10) | 55.0% |
| CoI-Contrarianism vs. Control | 2.63 (-1.32; 6.53) | 90.4% |
| **Psychosocial** | | |
| Entitlement vs. Control | -2.96 (-7.99; 2.16) | 87.5% |
| CoI-Contrarianism vs. Control | 3.37 (-1.88; 8.55) | 89.7% |
| ^a^ The median of the posterior distribution over linear effects, with 2.5% and 97.5% percentiles representing a compatibility interval (CI).  ^b^ The proportion of the posterior distribution over linear effects which is in the direction of the median. | | |
